# Case Report: Niraparib-Related Pulmonary Embolism During the Treatment of *BRCA* Mutant Advanced Ovarian Cancer

**DOI:** 10.3389/fonc.2022.853211

**Published:** 2022-03-10

**Authors:** Qiang Wei, Dong-Sheng Chen, Yuan-Hua Liu

**Affiliations:** ^1^Ultrasonic Department, Jiangsu Cancer Hospital and Jiangsu Institute of Cancer Research and The Affiliated Cancer Hospital of Nanjing Medical University, Nanjing, China; ^2^The State Key Laboratory of Translational Medicine and Innovative Drug Development, Jiangsu Simcere Diagnostics Co. Ltd., Nanjing, China; ^3^Department of Medical Oncology, Jiangsu Cancer Hospital and Jiangsu Institute of Cancer Research and The Affiliated Cancer Hospital of Nanjing Medical University, Nanjing, China

**Keywords:** Niraparib, pulmonary embolism, *BRCA* mutation, ovarian cancer

## Abstract

Niraparib, an oral, potent, highly selective poly (ADP-ribose) polymerase (PARP) inhibitor, has promising clinical benefit for maintenance treatment of patients with ovarian cancer in partial response to platinum-based chemotherapy, especially in patients with *BRCA* mutation. In publicly available niraparib treatment-related adverse events, gastrointestinal disorders and hematological toxicities were most commonly reported with manageable safety profile. Herein, we first describe a severe and never-reported pulmonary embolism (PE) associated with the use of niraparib in a patient with *BRCA* mutation advanced high-grade serous ovarian cancer and received anticoagulant therapy after PE. There have been no reports of PE caused by the use of niraparib in patients with advanced high-grade serous ovarian cancer; knowledge of the occurrence of PE after the use of niraparib may assist other clinicians in managing this rare but potentially serious toxic effect.

## Background

Niraparib, an oral, potent, highly selective poly (ADP-ribose) polymerase (PARP) inhibitor, has promising clinical benefit for maintenance treatment of patients with ovarian cancer in partial response to platinum-based chemotherapy, especially in patients with *BRCA* mutation or homologous recombination deficiency (HRD)-positive disease (1, 2). In publicly available niraparib treatment-related adverse events, the most common drug-related treatment-emergent adverse events were anemia and thrombocytopenia, and the most common treatment-emergent serious adverse events were small intestinal obstruction, thrombocytopenia and vomiting.^1^ Herein, we first describe a severe and never-reported PE associated with the use of niraparib in a patient with *BRCA* mutant advanced high-grade serous ovarian cancer.

## Case Presentation

**Figure 1:**
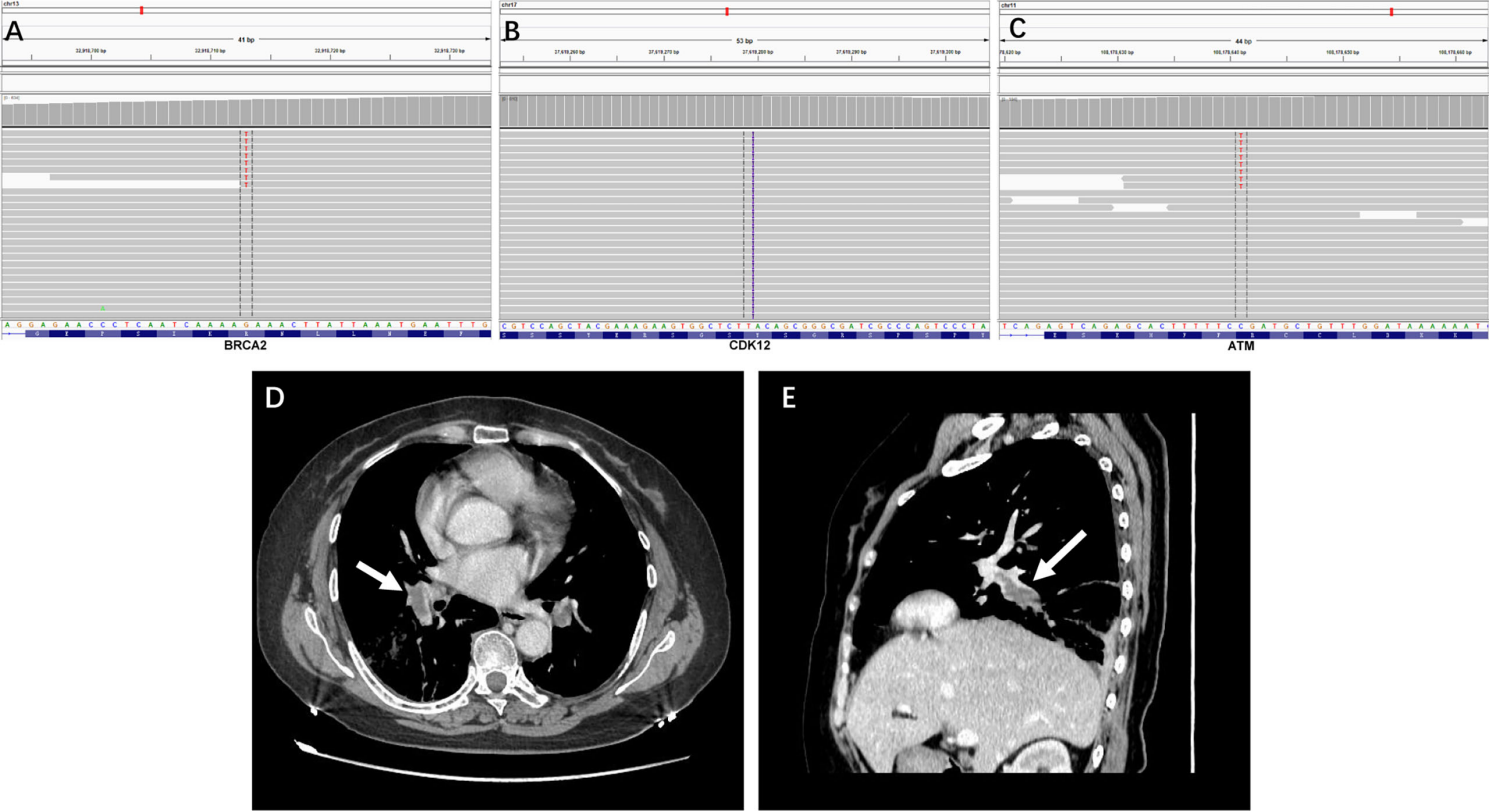
**(A–C)** The integrative genomics viewer snapshot of *BRCA2* c.6860G>T, *CDK12* c.956dupA, and *ATM* c.5692C>T mutation. **(D**, **E)** Contrast-enhanced CT revealed dilated pulmonary veins with hypodense filling defect **(E)**, 35.0 × 13.2 mm) highlighting the presence of thrombosis (arrow).

A 55-year-old female patient was diagnosed with advanced high-grade serous ovarian cancer after comprehensive multidisciplinary consultation. Platinum-based chemotherapy was administered as first-line treatment, and the disease was evaluated as partial response. To seek for an effective maintenance therapy, tumor *BRCA* mutation and HRD status were analyzed using a next-generation sequencing assay in patient providing tissue sample in a CAP-authenticated lab. Subsequently, *BRCA2* c.6860G>T (1.85% abundance), *CDK12* c.956dupA (22.04% abundance), and *ATM* c.5692C>T (1.64% abundance) mutations (**Figures 1A** **–C**) were detected and confirmed by polymerase chain reaction assay, suggesting PARP inhibitor may show promising clinical benefit for the maintenance treatment.

Two months after the ending of the last cycle of chemotherapy, niraparib (200 mg once daily) was administered for the following maintenance treatment; clinical and radiological follow-up within the first 2 months showed no evidence of progression and severe adverse reactions. In the second and a half month of taking niraparib, the patient was presented to the emergency room with the chief complaints of dyspnea, chest pain, and lower abdominal pain that had begun a few days earlier. On presentation, she was mild febrile and had tachycardia with a regular rate. Laboratory studies revealed activated partial thromboplastin time of 22.3 s (normal range (NR), 25.0 to 31.3 s); the serum D-dimer was 23.19 μg/ml (NR < 0.55 µg/ml); and the serum fibrinogen was 4.38 g/L (NR, 2–4 g/L). Other coagulation parameters such as prothrombin time and thrombin time were within normal ranges. Emergency contrast-enhanced computed tomography (CT) revealed dilated pulmonary veins with hypodense filling defect highlighting the presence of thrombosis (**Figures 1D, E**).

Based on the clinical presentation, CT images, and remarkably increased serum D-dimer and fibrinogen levels, a diagnosis of PE caused by the use of niraparib was made; she was then transported to the high care unit under anticoagulant therapy. Three days after the treatment, CT examination detected the remaining thrombi and her serum D-dimer decreased to 3.66 μg/ml. A week later, she was discharged and discontinued from the use of niraparib; no PE symptoms occurred on the follow-up treatment.

## Discussion

The standard of care for frontline therapy of advanced ovarian cancer is mainly platinum-based chemotherapy in some settings (3, 4). Although most patients respond to initial therapy, most of them will ultimately succumb to the disease. Usually, the recurrence of the disease poorly responds to first- and sometimes even second-line chemotherapy. In this scenario, it is possible that the ovarian cancer inherent resistance may be due to reduced immunosurveillance and drug-resistant cells (5, 6). PARP inhibitors are a new treatment approach for ovarian cancer and other cancers with underlying impaired DNA repair. For instance, PARP inhibitors also bring breakthrough progress in prostate cancer (7). The novel combinations of PARP inhibitors with other anticancer therapies, including inhibitors of VEGF, PD-1, or PD-L1, along with anti-CTLA 4 monoclonal antibodies, are well tolerated and clinically effective (8). In publicly available niraparib treatment-related adverse events, gastrointestinal disorders and hematological toxicities were most commonly reported with manageable safety profile.

PE is a serious complication of surgery and a leading cause of death in women with gynecologic cancer. Serous tubal intraepithelial carcinoma (STIC) is currently considered the precursor lesion of pelvic high-grade serous carcinoma. The incidence of STIC in BRCA1–2 variants was low (9). Early detection and adequate intervention are crucially important in order to prevent PE. In our case, the chief complaints of chest pain and lower abdominal pain suggested the possible presence of deep vein thrombosis. During the thrombosis process, the functions of the coagulation system, anticoagulation system, and fibrinolytic system are activated. D-dimer is a degradation product of fibrin, present in the blood when thrombi are broken down. The level of D-dimer reflects fibrin concentration and is known to have a high positive predictive value. In our case, remarkably increased fibrinogen levels and D-dimer levels suggested the rapid progression of blood coagulation and fibrinolysis. The contrast-enhanced computed tomography revealed dilated pulmonary veins with hypodense filling defect highlighting the presence of thrombosis. Chemotherapy is considered a significant risk factor for cancer-associated thrombosis and may have contributed to its increasing incidence because of the harm on the vascular epithelium (10). In view of niraparib being administered for the maintenance treatment 2 months after the ending of the last cycle of chemotherapy, this eliminated the possibility of the side effect from chemotherapy. Finally, based on the clinical presentation, CT images, and remarkably increased serum D-dimer and fibrinogen levels, a diagnosis of PE caused by the use of niraparib was made.

There are several reports of PE occurring after operation or during chemotherapy in patients with ovarian cancer (11–13). However, confirmed by publicly available niraparib treatment-related adverse events, there have been no reports of PE caused by the use of niraparib in patients with advanced high-grade serous ovarian cancer. In summary, we describe for the first time the occurrence of PE associated with the use of niraparib in a patient with *BRCA* mutant advanced high-grade serous ovarian cancer. Frankly speaking, we cannot give a detailed mechanism to explain the relationship between the use of niraparib and the occurrence of PE; we hypothesized that off-target inhibition of yet another ubiquitously expressed target may be responsible. Anyway, knowledge of the occurrence of PE after the use of niraparib may assist other clinicians in managing this rare but potentially serious toxic effect.

## Data Availability Statement

The datasets for this article are not publicly available due to concerns regarding participant/patient anonymity. Requests to access the datasets should be directed to the corresponding author.

## Ethics Statement

The studies involving human participants were reviewed and approved by the Jiangsu Cancer Hospital. The patients/participants provided their written informed consent to participate in this study. Written informed consent was obtained from the individual(s) for the publication of any potentially identifiable images or data included in this article.

## Author Contributions

We were all involved in the clinical care and management of the patient, collecting the data, and drafting the manuscript. QW and DC collected data and wrote the first draft of the manuscript. YL supervised the work. All authors critically reviewed and approved the final version.

## Conflict of Interest

Author DC was employed by Jiangsu Simcere Diagnostics Co. Ltd., Nanjing, China.

The remaining authors declare that the research was conducted in the absence of any commercial or financial relationships that could be construed as a potential conflict of interest.

## Publisher’s Note

All claims expressed in this article are solely those of the authors and do not necessarily represent those of their affiliated organizations, or those of the publisher, the editors and the reviewers. Any product that may be evaluated in this article, or claim that may be made by its manufacturer, is not guaranteed or endorsed by the publisher.
